# Microscopic and Microspectrophotometric Evaluation of Colour Changes in Cotton Fibres Exposed to Natural and Artificial Solar Radiation: Forensic Implications

**DOI:** 10.3390/polym18101178

**Published:** 2026-05-11

**Authors:** Jolanta Wąs-Gubała, Weronika Sarnowska, Bartłomiej Feigel

**Affiliations:** 1 Institute of Forensic Research, Westerplatte 9, 31-033 Krakow, Poland; bfeigel@ies.gov.pl; 2 Faculty of Chemistry, Jagiellonian University, Gronostajowa 2, 30-387 Krakow, Poland; weronika.sarnowska@student.uj.edu.pl; 3 Department C-1, Faculty of Chemical Engineering and Technology, Cracow University of Technology, Warszawska 24, 31-155 Krakow, Poland

**Keywords:** cotton, light exposure, colour change, UV–Vis microspectrophotometry, fluorescence microscopy, stereoscopic microscopy, colour difference (Delta E), forensic fibre analysis

## Abstract

The objective of this study was to evaluate colour changes in cotton fibres within knitted fabric structures under different light exposure conditions and to assess the applicability of forensic analytical methods for this purpose. Fabrics of three distinct colours were exposed to two types of irradiation: natural sunlight and artificial light in a controlled climatic chamber. A multi-scale analytical approach was applied, including visual inspection and stereomicroscopy for macro-level evaluation, followed by bright-field microscopy, fluorescence microscopy, and UV–Vis microspectrophotometry for single-fibre characterisation. Visual assessment of fabrics revealed perceptible colour differences between exposed and unexposed samples, whereas stereomicroscopy did not consistently enhance the detection of these alterations. Bright-field and fluorescence microscopy showed no visually perceptible differences between fibres from exposed and unexposed fabrics of the same colour. Microspectrophotometric measurements did not reliably capture colour changes in single cotton fibres, particularly in samples exposed to natural sunlight. Furthermore, total colour difference (ΔE) values, ranging from 0.248 to 6.652, were found to be unreliable at the single-fibre level due to significant spatial variability across different measurement sites. The findings indicate that, while light exposure may induce perceptible colour alterations in cotton knitted fabrics, the forensic examination of single fibres does not necessarily reflect these macro-scale changes. From a forensic perspective, the stability of microscopic and microspectrophotometric characteristics supports reliable fibre comparison, even after post-event exposure to sunlight.

## 1. Introduction

The colour fastness of dyed cotton knitwear exposed to solar radiation is a key factor influencing both the material’s performance and its relevance in forensic fibre analysis. In forensic examinations involving microtraces in the form of coloured cotton fibre fragments, the primary parameter subject to analytical verification is colour [1,2]. This attribute is fundamentally associated with the physical principles of electromagnetic radiation and the biological and psychophysical mechanisms of human vision.

Colour is perceived as a result of the interaction between electromagnetic radiation in the visible spectrum (approximately 380–780 nm) and a material surface, followed by the detection of reflected or transmitted stimuli by the human visual system [3]. The perceived colour of an object arises from the selective absorption of incident light and the reflection or transmission of non-absorbed wavelengths, depending on whether the material is opaque or transparent. Consequently, a material’s colour may be spectrally simple or complex, typically comprising multiple spectral components. While electromagnetic radiation absorption is not limited to the visible spectrum and often extends into the ultraviolet (UV) and infrared (IR) regions, only absorption within the visible range directly determines the perceived colour [4].

In cotton fibres, colouration is primarily achieved using reactive dyes, i.e., chemical compounds that strongly absorb visible light and form covalent bonds with cellulose, resulting in durable and vibrant colouration [5,6]. Their chromophores are specifically engineered to absorb within the visible spectrum, enabling efficient and permanent dyeing of the substrate.

The primary factors influencing the ageing of textile materials, aside from environmental contributors such as acid rain and general air pollution, are moisture, temperature, and the intensity of solar radiation. Among these, ultraviolet radiation, particularly in the UV-A and UV-B ranges, has been identified as the most damaging to fibre structure and colour stability [7,8,9].

The colour fastness of dyed materials under exposure to daylight is evaluated according to the International Organization for Standardization method ISO 105-B01 [10]. This standard outlines several comparative test procedures designed to assess colour change. The primary methods specified include evaluation based on the grey scale for colour change and the blue wool scale. The standard also provides all necessary technical details, such as the required sample dimensions and the testing conditions under which the assessments must be conducted. While these traditional scales rely on visual assessment, modern colourimetry often quantifies this change using the ΔE parameter, which measures the total colour difference in the CIELab colour space. According to the ISO 105-A05 standard [11], these values correlate with specific levels of perceived change: a ΔE of 0–0.2 indicates no perceptible change, a value of approximately 1.7 represents a slight but noticeable change, while values exceeding 6.8 signify a severe loss of colour. A clear relationship can be observed: the lower the ∆E value calculated according to the CIELab formula, the greater the resistance of the material to colour change.

However, visual assessment of colour change is neither as precise nor as objective as instrumental measurement. The acceptance of visual methods by ISO standards may be attributed to the general simplicity and accessibility of such testing. These tests do not require specialised equipment, are quick and easy to perform, and typically do not involve prior sample preparation. ISO 105-B01 [10] also acknowledges that, during sunlight exposure, colour change in textile materials may not only manifest as fading, but also as a shift in hue—for example, from yellow to brown. Such transformations cannot be accurately represented by the grey scale or the blue wool scale. Detecting these types of changes relies on the observer’s ability to recognise and record them during the visual evaluation process.

Solar radiation is known to initiate oxidative and photo-induced reactions in dyed fibres, leading to fading, hue shifts, or loss of chromatic intensity [12]. The extent of these changes depends on fibre composition, dye type, dyeing method, and exposure conditions. In forensic investigations, discoloured fibres may reduce the accuracy of comparisons between evidence and reference materials, particularly when human visual assessment is involved. Accordingly, it is pertinent to assess the detectability of these changes through the application of advanced optical microscopy and microspectrophotometry [13,14].

Scientific research in forensic sciences is based on data reflecting real crime scene conditions. Therefore, when investigating the effect of sunlight on textiles, optimal environmental conditions were first identified. According to the 2023 Meteorological Yearbook (IMGW-PIB), based on data from 55 meteorological stations across Poland, a continued increase in the number of sunny days and the average annual temperature has been observed [15]. In 2023, the national average temperature reached 10.1 °C, exceeding values from the previous two years. The highest temperature (35.5 °C) was recorded in August in Kętrzyn, and the lowest (−16.7 °C) in December in Racibórz-Studzienna. Despite being classified as a warm year, considerable monthly fluctuations occurred. Atmospheric humidity, which affects both the human body and textiles, varies with season and location. Although long-term humidity levels remain relatively stable, rising global temperatures are associated with increased atmospheric humidity.

In recent years, only a few studies have been conducted on the sunlight resistance of textile materials in the context of Poland’s specific climatic conditions. However, these findings are highly relevant to the broader region of Europe, which shares a similar temperate transitional climate characterised by significant seasonal fluctuations in solar radiation and humidity. An example study examined both colour stability and mechanical durability, including tear resistance, by exposing fabrics outdoors on a terrace surface for over 1200 h [16]. The materials were subjected to continuous natural weathering, involving various environmental factors beyond solar radiation. All tested samples exhibited noticeable colour changes.

Another study focused on protective fabrics used in firefighting garments, which underwent accelerated ageing in a climate chamber equipped with a xenon arc lamp to simulate UV exposure [17]. After 200 h of irradiation, the samples were tested for mechanical strength. The results confirmed a significant deterioration in the mechanical properties due to UV exposure.

The aim of the study presented in this paper is to investigate the effects of both natural and artificial sunlight exposure on the colour stability of cotton knitting fabrics and fibres dyed in three distinct colours. Environmental variables under natural sunlight, such as radiation intensity, exposure duration, temperature, humidity, wind, and textile morphology, change continuously over time, whereas in climatic chamber experiments these parameters are controlled and kept constant to ensure reproducible conditions [18,19]. A combination of different methods of optical microscopy and UV–Vis microspectrophotometry (MSP) was employed to evaluate potential colour changes occurring in the fibres. This research seeks to enhance understanding of sunlight-induced colour changes of cotton textiles, highlighting its implications for both forensic fibre analysis and the clothing industry. Furthermore, the study underscores the importance of accounting for environmental exposure in the forensic interpretation of fibre evidence and in the development of more light-stable textile materials.

## 2. Materials and Methods

### 2.1. Material Characterisation

The research material consisted of textile samples provided by MIRWAL^®^ (Sieradz, Poland). The samples were single-jersey knitted fabrics made of 100% cotton, with a comparable areal density of approximately 220 g/m^2^. All fabrics were dyed using the same class of colourants, i.e., reactive dyes, resulting in three different initial colours: black (designated as 1B), mint (2M), and orange (3O).

Prior to the dyeing process, the fabrics underwent biopolishing, a wet treatment involving the application of specific enzymes under acidic conditions and elevated temperatures. This process effectively removes protruding fibres from the fabric surface. At the final stage of sample preparation, the 3O fabric sample was subjected to a dye fixation process involving the application of a quaternary ammonium compound. Detailed information regarding the specific reactive dyes used, as well as the exact preparation procedures, was not disclosed by the manufacturer due to commercial confidentiality.

### 2.2. Experimental Procedure Involving Solar Radiation

#### 2.2.1. Under Natural Conditions

Fabric swatches measuring 10 × 15 cm were prepared from each of the three knitted fabric variants and were stitched onto a base fabric made of white cotton. The prepared samples were then exposed to natural sunlight by hanging them outside a window. Exposure took place exclusively on sunny days during the autumn–winter period in Poland, from 17 October 2024 to 7 March 2025. The initial and final air temperature, relative humidity, and daily UV index were monitored (the “initial” value was recorded at the time of placing the samples outside, while the “final” value was recorded upon their removal). Temperature and humidity readings were obtained using the “Pogoda” weather application (version 1.6.41.10) installed on an Android device. UV index values were recorded from the website https://www.pogodairadar.pl/indeks-uv/krakow/6754153 (accessed on 7 March 2025). Fabric fragments (5 × 10 cm) were sampled at 100-h intervals of sunlight exposure until a cumulative irradiation time of 300 h was reached. This duration was attainable within the specified autumn–winter period. The collected subsamples were protected from any additional light and humidity exposure.

#### 2.2.2. Under Artificial Conditions

Each of the three differently coloured cotton knitted samples was stitched onto white filtration paper. The samples, measuring 30 × 5 cm, were placed in a KBF 260 climate chamber (BINDER GmbH, Tuttlingen, Germany). The irradiation conditions were set as follows: a temperature of 35.5 °C and a relative humidity of 70%. These parameters were chosen to simulate extreme weather conditions recorded in Poland in 2023 [15].

Subsamples, measuring 4 × 5 cm, were collected at regular intervals (2 days) throughout the 10-day exposure period. After collection, the subsamples were shielded from further exposure to light and humidity.

### 2.3. Instrumental Methods Characterisation

#### 2.3.1. Microscopic Methods

Stereomicroscopy was performed using a Leica MZ16 stereomicroscope (Leica Microsystems, Wetzlar, Germany) equipped with a Digital Sight DS-Fi3 high-definition colour microscope camera (Nikon, Sendai, Japan). This setup served to observe and record colour changes in the knitted samples, as well as to prepare specimens for subsequent instrumental analysis. Data processing was carried out using the NIS-Elements D software suite (Version 5.30.00, Nikon, Sendai, Japan).

Fibre fragments extracted from the examined knitted fabrics, both exposed and unexposed, were mounted on microscope glass slides and a cover slip (Menzel Glaser, Braunschweig, Germany), in pure glycerine (Sigma-Aldrich, Burlington, MA, USA; refractive index 1.474). More detailed optical observations of the fibres were conducted using bright-field and fluorescence microscopy with a Leica DM 2700 P research microscope (Leica Microsystems, Wetzlar, Germany) at magnifications of 100×, 200×, and 400×. Fibres from unexposed coloured knitted cotton samples were compared with those subjected to 100, 200, and 300 h of sunlight exposure, as well as 10 days in a climate chamber. The fibres selected for analysis were taken from the outermost parts of the threads, i.e., those most exposed to light radiation. Images acquired under transmitted white light and with fluorescence filter blocks for ultraviolet, violet, blue, and green light were analysed, maintaining identical illumination conditions throughout the observation of each sample. The shade and depth of dyeing, fibre morphology, and fluorescence properties were examined.

#### 2.3.2. UV–Vis Microspectrophotometry

Absorption spectra were obtained using a 20/30PV PRO™ microspectrophotometer (CRAIC Technologies, San Dimas, CA USA), equipped with a Zeiss Ultrafluar 40× objective (Carl Zeiss Microscopy GmbH, Jena, Germany). Measurements were performed in the UV–Vis region (200–800 nm); however, in order to evaluate colour change, selected analysis of experimental fibres focused exclusively on the visible range (420–800 nm). This also results from the fact that forensic practice indicates that when the device operates over its full working range (200–800 nm), the risk of cotton fibres discolouring as a result of the measurement increases. The measurement parameters were as follows: an aperture size of 6.0 × 6.0 µm, 25 scans averaged, and a spectral resolution of 1 nm.

MSP specimens were collected from experimental samples, both unexposed and exposed to natural sunlight for 100, 200, and 300 h, as well as from samples irradiated in a climate chamber for 10 days. Fibre fragments were mounted on quartz microscope glass slides and a cover slip (CRAIC Technologies, USA), in pure glycerine (Sigma-Aldrich; refractive index 1.474). For each MSP specimen, ten measurements were taken from different fibres along a single yarn.

In addition to these measurements, preliminary studies were conducted on coloured fibres from the original (unexposed) fabrics. These assessed the effect of radiation generated by the microspectrophotometer’s deuterium and halogen lamps on the results, as well as intra-yarn colour variation.

Reference spectra were collected adjacent to each fibre prior to its measurement. All fibres were measured in the same orientation (north–south) based on published methodologies [1,2]. A mean spectrum was calculated for each set of measurements. All analyses and calculations, including evaluation of spectral profile, as well as colourimetric analysis, were performed using LambdaFire™ 2.0 software and CRAIC ColourPro™—colour coordinate analysis application. Colour coordinates were measured in the CIE L*a*b* colour space, and the total colour difference (ΔE) was calculated using the CIE76 formula. Prior to this analysis, the recorded absorption spectra were converted into transmittance spectra to ensure compatibility with the colourimetric calculation software (LambdaFire™ 2.0 software).

## 3. Results

### 3.1. Microscopic Observations of Colour Variations in Samples and Their Fibres

**Figure 1:**
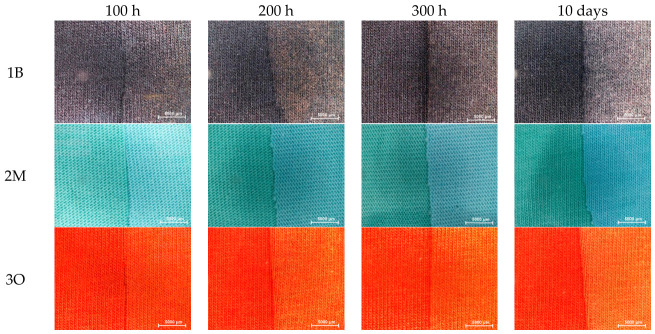
Stereomicroscope comparisons of unexposed knitted fabric samples (left sides of the comparisons) alongside samples exposed to natural sunlight for 100, 200, and 300 h, and samples irradiated in a climate chamber for 10 days (right sides of the comparisons).

The stereoscopic images of the original knitted fabric samples, which were not exposed to solar radiation, were compared with those of samples collected after 100, 200, and 300 h of natural sunlight exposure, as well as after 10 days of artificial irradiation (Figure 1).

Among the tested samples, Samples 1B and 3O exhibited the most pronounced colour changes, specifically significant fading, after 10 days of exposure in the climate chamber. Solar radiation also induced a progressive fading effect, which was particularly pronounced in Sample 3O as the exposure time increased. Sample 2M showed a significant colour change relative to the unexposed sample as early as after the first 100 h of sunlight exposure, shifting from mint to light blue. This process continued throughout the duration of the irradiation experiment for this sample. It should be emphasised that all colour differences in the dyed cotton knitted fabrics were much more pronounced when observed with the naked eye under natural light illumination. Stereomicroscopic images, and consequently microphotographs taken during microscopic observation, tend to flatten and diminish the perceived differences.

Despite prolonged exposure and the colour variations observed in the knitted fabrics under the stereomicroscope, bright-field and fluorescence microscopy of single fibres revealed no significant morphological or colour changes. Regardless of the exposure type or duration, the microscopic appearance of the fibres remained consistent with the unexposed control samples. Any observed differences were below the threshold of visual detection. While bright-field and fluorescence microscopy allowed for clear differentiation between the original fibre colours, they did not reveal any significant fading or colour shifts throughout the experiment.

### 3.2. Evaluation of Colour Variations in Single Fibres Using UV–Vis Microspectrophotometry

#### 3.2.1. Preliminary Study of Unexposed Samples and Their Colour Variability

**Figure 2:**
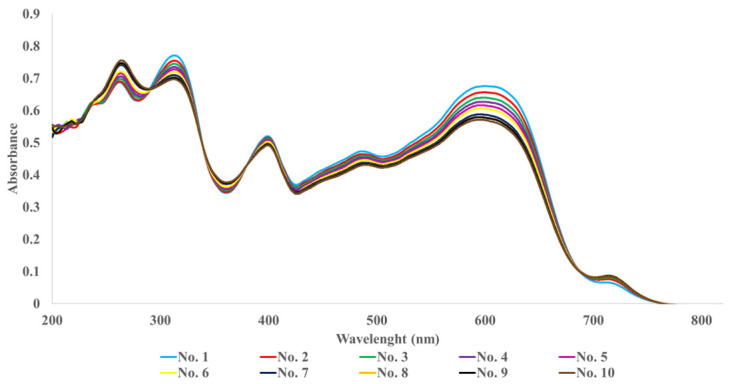
Comparison of ten replicate microspectrophotometric measurements recorded on the same fibre area of Sample 1B in the UV–Vis region (200–800 nm).

A series of ten microspectrophotometric measurements was conducted on the same fibre area to assess the influence of the irradiation source on colour fastness. Figure 2 illustrates the variation in absorbance intensity observed across ten consecutive measurements for a representative specimen (Sample 1B).

In all unexposed control samples analysed in this manner, colour changes were negligible. Although forensic fibre examination protocols recommend limiting the number of measurements, as repeated exposure to the microspectrophotometer’s light source may induce localised degradation, this effect was found to be minimal in the present study.

**Figure 3:**
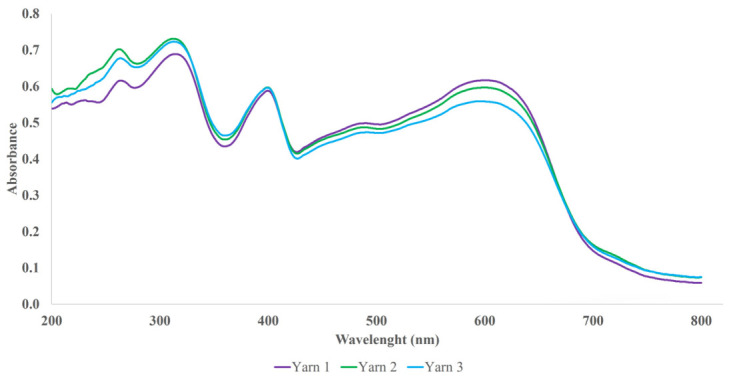
Comparison of mean spectra calculated from ten replicate microspectrophotometric measurements of fibres from three representative yarns of Sample 1B in the UV–Vis region (200–800 nm).

To assess the variability in fibre colouration within a single yarn, fibres from three individual yarns of each unexposed colour variant were examined. Figure 3 presents the mean spectra obtained for fibres across three separate yarns of representative Sample 1B.

For all examined samples, minor variations in dyeing intensity can be observed among the fibres within individual yarns, while the spectral profile, i.e., the shape of the absorption spectrum, remains consistent across all measurements. Consequently, the dye is considered to be uniformly distributed along the entire length of the yarn.

For each of the examined unexposed coloured cotton fibre samples, characteristic absorption peaks were observed in both the visible and ultraviolet regions. Although the UV region exhibits intense absorption, the visual assessment of the spectra focuses mainly on the visible range to enable direct correlation of spectral changes with visually perceivable fading.

The comparison of the mean spectra for each sample revealed distinct differences among the investigated cotton colours. Each dye/dyes produced a unique spectral profile, which, in practice, facilitates the effective differentiation of dyed fibres (a capability of significant importance in routine comparative forensic fibre examinations).

#### 3.2.2. Study of Exposed Fibre Samples and Their Colour Variability

Visual assessment of the mean spectra from all irradiated 1B samples indicates that the intensity did not change as anticipated, although a decrease in intensity relative to the untreated sample was expected. An overlap of the spectrum of the untreated sample and that of the sample exposed to solar radiation for 200 h can be observed. Similarly, the sample conditioned in the climatic chamber for 10 days exhibits a spectrum comparable to that of the sample exposed for 300 h. In contrast, the spectrum of the sample exposed for 100 h shows a significantly higher intensity, which is inconsistent with the initial assumptions. Regardless of the intensity of variations observed between successive measurements, the spectral profile remained unchanged, and no shifts in peak positions were detected within the visible light range. This indicates that the fibre colour can still be detected and identified irrespective of the duration of experimental irradiation. The ΔE values measured for fibres from each irradiated 1B sample relative to the untreated sample are presented in Table 1.

Overall, Sample 1B exhibited minimal colour changes throughout the entire exposure period. ΔE values ranged from 0.248 to 1.525, with the largest change observed after 100 h, corresponding to a slight increase in lightness and a minor chromatic shift toward the yellow region. In most cases, these changes are below the visual perception threshold, indicating excellent colour stability.

The statistical analysis of ΔE values, calculated relative to the mean spectra of cotton fibres from Sample 1B, is presented in Table 2. The analysis includes the standard deviation (SD) and the coefficient of variation (%CV), providing insight into the variability and repeatability of the measurements under both natural and artificial ageing conditions. The SD values ranged from 1.67 to 4.57, while the CV values varied between 44.69% and 92.52%. The relatively high CV values indicate considerable relative variability, which may be attributed to the low absolute ΔE values and their sensitivity to minor spectral fluctuations.

Sample 2M yielded particularly interesting results, as distinct colour shifts were visually apparent throughout the experiment. However, a comparison of the MSP spectra reveals that these changes did not follow a uniform trend. The unexposed sample exhibited the lowest absorbance intensity, with relatively large deviations observed between subsequent intervals; nevertheless, all recorded intensities remained notably low. Interestingly, the unexposed sample and the sample subjected to 10 days of artificial ageing showed the highest spectral correlation, which contradicts the visual observations. Consistent with the other samples, the spectral profile remained stable despite fluctuations in intensity. This finding is particularly unexpected, as the sample underwent a visible chromatic shift from mint green to light blue. Table 1 presents the results of ΔE measurements for fibres from each sample exposed to radiation, relative to the unexposed sample, which ranged from 1.802 to 6.652. The most pronounced colour change was observed for the sample exposed to natural sunlight for 200 h, reflecting a significant increase in lightness and chromatic shifts toward the green and blue regions.

The statistical evaluation of ΔE values for Sample 2M, calculated relative to the mean spectra of cotton fibres, is summarised in Table 2. The analysis comprises the standard deviation (SD) and the coefficient of variation (%CV), which together describe the dispersion and relative variability of the measurements under both natural and artificial ageing conditions. The SD values ranged from 0.73 to 2.08, while the CV values varied between 45.54% and 81.83%. In comparison to the other samples, these results indicate generally lower absolute variability, while the CV values remain elevated due to the low magnitude of ΔE and its sensitivity to minor spectral changes.

Similar intensity levels were observed in the spectra of all irradiated 3O samples; however, as with the other samples, no consistent trend in the intensity changes in single fibres could be identified. The lowest intensity was noted for the unexposed sample, followed successively by the samples exposed for 100 h, 200 h, and 300 h. This progression aligns with both theoretical expectations and visual observations. As with the other samples, the spectral profile remained unchanged despite the irradiation. Table 1 presents the results of ΔE values for fibres from Sample 3O exposed to radiation, relative to the unexposed sample. These values ranged from 0.454 to 1.423 and are remarkably low, indicating high colour stability. The most pronounced difference was observed after 300 h of natural sunlight exposure, characterised by minor chromatic shifts toward the green and blue regions, along with a slight decrease in lightness.

The statistical analysis of ΔE values, calculated relative to the mean spectra of cotton fibres from Sample 3O, is presented in Table 2. The analysis includes the standard deviation (SD) and the coefficient of variation (%CV), providing insight into the variability and repeatability of the measurements under both natural and artificial ageing conditions. The SD values ranged from 1.30 to 5.42, while the CV values ranged from 49.02% to 107.48%. The relatively high CV values indicate considerable relative variability, which may be attributed to the low absolute ΔE values and their sensitivity to minor spectral fluctuations.

## 4. Discussion

The type of dye may significantly influence photodegradation and colour fading, as reported in the literature [19]. However, in the present study, the exact chemical composition of the dyes used in the investigated textile fibres is unknown, as the materials were commercially sourced and no information regarding dye formulation was provided by the manufacturer. Therefore, it was not possible to analyse the effect of specific dyes on the degradation process. The investigation was limited to comparing the colour changes in the fibres during exposure, which allows evaluation of overall photodegradation behaviour without attributing it to particular dye types.

Colour fading may also result from aerial oxidation of dyes in the presence of atmospheric oxygen, which promotes oxidative cleavage of chromophoric groups and degradation of dye molecules, as reported in recent studies [20]. In a forensic context, however, this remains a challenging parameter to control, as the specific chemical identity and concentration of the dyes used in textile evidence are typically unknown during the investigation.

Preliminary visual inspection of the knitted fabric samples revealed noticeable colour shifts following experimental irradiation compared to the unexposed reference materials. This effect was evident in samples subjected to both natural solar radiation and artificial irradiation in a climatic chamber. The colour change was more pronounced in the artificially irradiated samples, which can be attributed to the controlled and stable experimental conditions, specifically the constant elevated temperature and relative humidity maintained throughout the process [18].

During microscopic observation under bright-field illumination, certain experimental fibres exhibited variations in colour intensity; however, no systematic trend correlating with increased irradiation time was identified. These fluctuations were not universal across all examined materials and did not influence the final outcomes obtained via fluorescence microscopy. Such differences likely stemmed from the spatial positioning of individual fibres within the knitted structure or from inherent variations in fibre morphology and thickness. In knitted fabrics, the yarn follows an alternating path over and under the fabric surface; consequently, certain segments are subjected to different radiation intensities than others [21,22]. Furthermore, the non-uniform cross-sectional shape and natural convolutions of cotton fibres can significantly affect light attenuation during bright-field and fluorescence microscopy observation, leading to perceived fluctuations in colour intensity [1].

Microspectrophotometric analysis in the visible and ultraviolet range (MSP) constitutes an excellent tool for comparing the colouration of single fibres [1,23]. However, the presented studies demonstrate that MSP may not provide a sufficiently reliable method for quantifying colour changes in cotton fibres exposed to natural and artificial sunlight. The intensity of absorption peaks characteristic of the dyes rarely shifted in a manner consistent with the visual degradation observed in the knitted fabrics. These measurements are further influenced by the specific selection of the sampling area; for instance, whether the analysed region was fully exposed to radiation or shielded by overlapping fibres within the yarn structure. Furthermore, inherent variations in fibre thickness, microstructure, and the presence of natural micro-imperfections, often imperceptible under standard optical microscopy, significantly impact the spectral data.

The investigated samples displayed varying degrees of colour stability throughout the experimental period. Among the tested specimens, Sample 1B exhibited excellent photostability under all conditions, with negligible deviations in its chromaticity coordinates. In contrast, Sample 2M proved highly susceptible to colour alterations, undergoing significant and irreversible changes in visual appearance. Finally, Sample 3O demonstrated intermediate behaviour, characterised by only minor and transient colour shifts.

Among the studied materials, Sample 3O showed the highest relative dispersion, while Sample 3M generally exhibited the lowest absolute variability. Overall, the results confirm good colour stability of the cotton fibres under both natural and artificial ageing conditions.

The use of visual comparison methods in studies conducted according to ISO 105-B01 was considered justified [10]. Nevertheless, the measurement of the ΔE parameter as an analytical and objective indicator remains a useful approach for eliminating human factors and improving measurement precision and accuracy. However, this parameter provides reliable results only when assessing entire fabrics, whereas, as shown by the results of the conducted experiment, the measurement of ΔE values for single fibres using microspectrophotometry cannot be regarded as a dependable evaluation. The tested fabrics were made from natural fibres and showed uneven colouration; therefore, measurements on individual fibres did not reflect the actual changes that occurred in the materials during the experiment [24]. In particular, for the measurement of the ΔE parameter in samples exposed to natural sunlight, the values did not always correspond to the expected results. This discrepancy may be attributed to the difficulty of ensuring a specific measurement site was consistently exposed to sunlight, as fibre samples might have been taken from unevenly irradiated areas. Conversely, this type of experiment provides a closer simulation of the real-life conditions experienced by clothing in an outdoor environment. In studies conducted in textile laboratories, when using reflectance spectrophotometry, the sample must be sufficiently large, which reduces the risk of analysing unexposed fragments. In contrast, the exposure surface of samples in the climatic chamber was more precisely controlled.

From a forensic perspective, visual assessment or stereomicroscopy of textile materials can be misleading, as perceptible colour shifts might suggest different origins for the compared materials. However, the results of this study indicate that microscopic analysis of single fibres, particularly via fluorescence microscopy, yields more robust results. The experimentally treated fibres exhibited the same fluorescence characteristics as the unexposed samples across all examined spectral ranges, even after prolonged and intense light exposure. This stability is critical in forensic casework, as it prevents the erroneous exclusion of fibres that may have originated from the same source but underwent photodegradation, for instance, during the interval between a forensic event and the collection of evidence.

Microspectrophotometric analysis of the fibres showed that, even after prolonged exposure to both natural solar radiation and artificial radiation in a climatic chamber, the spectral characteristics of the cotton fibres did not undergo significant alterations, thereby facilitating accurate forensic interpretation. A recognised limitation of this approach is the multitude of external factors that may influence the colour of worn garments, such as laundering, perspiration, soiling, or mechanical creasing, all of which can result in non-uniform colouration. While these variables were strictly controlled within the parameters of the present study, they represent critical considerations for the evaluation of real-world forensic evidence.

## 5. Limitations of the Study

The degradation of textile fibres is not a uniform process. Morphological variations, such as the ratio of crystalline to amorphous regions within the fibre-forming polymer, significantly influence the diffusion of oxygen and the localisation of degradation products. In forensic samples, the prior history of the textile (e.g., mechanical wear, laundering, or previous environmental exposure) is often unknown, which may introduce structural variables that are difficult to isolate.

A primary limitation in forensic fibre examination is the lack of manufacturer provenance regarding the specific chemical dyes and functional additives integrated during production. In forensic casework, the analyst is confronted with ‘unknown’ samples where the chemical identity of the chromophores is rarely disclosed or easily identifiable due to trace amounts of evidence. Since different chromophores exhibit varying degrees of photostability and distinct mechanisms of oxidative cleavage, this lack of chemical control inherently limits the precision of fading correlations and the ability to establish a chronological timeline of exposure for evidentiary material.

Furthermore, the non-linear relationship between dye loss and mechanical strength loss remains a challenge for comprehensive degradation modelling, and this issue often falls outside the scope of practical forensic science, where sample quantity is typically limited and destructive testing must be minimised.

## 6. Future Considerations

The study was conducted during the autumn–winter period. The natural light exposure experiment was therefore carried out only under conditions with low UV intensity, which may not fully reflect the pattern of fibre colour changes under strong sunlight. In future research, attention should be given to other types of materials, such as synthetic fibres, as well as to additional exposure parameters. In particular, it would be valuable to investigate the rate of colour change in seasonal textiles, such as summer fabrics, which are exposed to significantly higher UV index levels and prolonged solar radiation.

To overcome the limitation of unknown dye identities in trace evidence, future studies may focus on integrating Surface-Enhanced Raman Spectroscopy (SERS) in mapping mode. This technique could allow for the identification of specific chromophores within a single fibre without consuming the sample, enabling more precise degradation models tailored to specific chemical classes.

The lack of manufacturer data can be mitigated by creating comprehensive, open-access databases of degradation profiles for common commercial textiles. By cataloguing how specific fibre-dye combinations (e.g., reactive-dyed cotton vs. disperse-dyed polyester) react to controlled UV exposure, forensic experts could use comparative analysis to estimate exposure timelines more accurately.

Future research may employ other advanced chemometric tools, such as Principal Component Analysis (PCA) or Machine Learning algorithms, to analyse multi-dimensional data (colour, morphology, and chemical markers). This could help identify non-linear correlations between environmental factors and fibre degradation, even when the exact initial state of the textile is unknown.

## 7. Conclusions

The objective of this study was to evaluate the colour alterations in cotton fabrics and individual fibres resulting from light exposure. Two distinct irradiation methods were employed: natural sunlight and artificial irradiation within a climatic chamber. To ensure strictly controlled conditions, external variables such as laundering, drying cycles, and environmental factors (e.g., precipitation) were eliminated. The findings provide critical insights for forensic practice, demonstrating the extent to which light exposure can modify textile evidence over time. Furthermore, this research highlights the necessity of accounting for environmental degradation when analysing textile materials in the context of criminal investigations.

Stereomicroscopy proved less effective at assessing colour alterations in the knitted fabrics, whereas direct visual observation provided more detailed information regarding perceived colour variation. Bright-field and fluorescence microscopy, while highly effective for discriminating fibres with different initial colouration, did not reveal significant differences between exposed and unexposed fibres in this study. The research findings indicated that although samples could be distinguished by their base colour, subsequent irradiation of fabrics of similar hues did not consistently produce discernible changes in the microscopic images of their constituent fibres.

Although microspectrophotometric (MSP) measurements in the visible and ultraviolet ranges are effective for comparing the colouration of individual fibres, this study proved insufficiently reliable for quantifying change in cotton fibres exposed to natural and artificial sunlight. From a forensic perspective, this is a highly favourable outcome, as it prevents the erroneous exclusion of fibres originating from the same source that may have undergone varying degrees of light exposure following a forensic event.

## Figures and Tables

**Table 1:** Total colour differences (ΔE) of cotton fibres from Samples 1B, 2M, and 3O after natural sunlight exposure (100, 200, and 300 h) and artificial irradiation in a climate chamber (10 days).

Colour Difference Relative to the Unexposed Reference (ΔE)			
Sample	1B	2M	3O
100 h	1.525	5.942	0.454
200 h	0.248	6.652	0.468
300 h	0.639	4.960	1.423
10 days	0.658	1.802	0.603

ΔE represents the total colour difference calculated as the Euclidean distance between the L*a*b* coordinates.

**Table 2:** Statistical parameters (standard deviation and coefficient of variation) of ΔE relative to the mean spectrum of cotton fibres from Samples 1B, 2M, and 3O after natural sunlight exposure (100, 200, and 300 h) and artificial irradiation in a climate chamber (10 days).

Statistical Parameters of ΔE Relative to the Mean Spectra						
Sample	1B		2M		3O	
	SD	%CV	SD	%CV	SD	%CV
100 h	3.18	50.41	0.73	45.54	1.74	75.37
200 h	1.67	44.69	1.03	47.13	3.33	72.21
300 h	3.27	48.14	2.08	81.83	5.42	107.48
10 days	4.57	92.52	1.50	64.24	1.30	49.02

SD represents standard deviation, and %CV represents the coefficient of variation.

## Data Availability

The original contributions presented in this study are included in the article. Further inquiries can be directed to the corresponding author.

## Abbreviations

The following abbreviations are used in this manuscript:

MSP	Microspectrophotometry
ΔE	Delta E parameter

## References

[1] Robertson J., Roux C., Wiggins K. (2018). Forensic Examination of Fibres.

[2] ENFSI (2022). Best Practice Manual for the Forensic Examination of Fibres.

[3] French K., Rose K., Thompson I., Cornell E. (2008). The Evolution of Colour Vision Testing. Aust. Orthopt. J..

[4] Raluca B. (2016). A Review of Colour Measurements in the Textile Industry.

[5] Śmigiel-Kamińska D., Wąs-Gubała J., Stepnowski P., Kumirska J. (2020). The Identification of Cotton Fibres Dyed with Reactive Dyes for Forensic Purposes. Molecules.

[6] Khatri A., Peerzada M.H., Mohsin M., White M. (2015). A Review on Developments in Dyeing Cotton Fabrics with Reactive Dyes for Reducing Effluent Pollution. J. Clean. Prod..

[7] Saha B., Saha A., Das P., Kakati A., Banerjee A., Chattopadhyay P. (2024). A Comprehensive Review of Ultraviolet Radiation and Functionally Modified Textile Fabric with Special Emphasis on UV Protection. Heliyon.

[8] Kocić A., Bizjak M., Popović D., Poparić G.B., Stanković S.B. (2019). UV Protection Afforded by Textile Fabrics Made of Natural and Regenerated Cellulose Fibres. J. Clean. Prod..

[9] Malek T.N.M., Ibrahim N.A., Ibrahim N., Abd Hamid N.H. (2023). Cotton Woven Fabrics as Protective Polymer Materials against Solar Radiation in the Range of 210–1200 nm. Polymers.

[10] (2014). Textiles—Tests for Colour Fastness—Part B01: Colour Fastness to Light: Daylight.

[11] (1997). Textiles—Tests for Colour Fastness—Part A05: Instrumental Assessment of Change in Colour for Determination of Grey Scale Rating.

[12] Periyasamy A.P., Vikova M., Vik M. (2017). A Review of Photochromism in Textiles and Its Measurement. Text. Prog..

[13] Goodpaster J.V., Liszewski E.A. (2009). Forensic Analysis of Dyed Textile Fibres. Anal. Bioanal. Chem..

[14] Hu C., Mei H., Guo H., Zhu J. (2020). Colour Analysis of Textile Fibres by Microspectrophotometry. Forensic Chem..

[15] Institute of Meteorology and Water Management—National Research Institute (IMGW-PIB) (2023). Meteorological Yearbook 2023.

[16] Łężąk K. Aging of Textile Materials Intended for Protective Clothing under the Influence of Solar Radiation and Atmospheric Factors. http://archiwum.ciop.pl/25666.html.

[17] Kamocka-Bronisz R., Bronisz S. (2018). Impact of Solar Radiation on the Resistance of External Textile Used in Special Firefighter Clothing. Zesz. Nauk. SGSP.

[18] Friedrich D. (2018). Comparative study on artificial and natural weathering of wood-polymer compounds: A comprehensive literature review. Case Stud. Constr. Mater..

[19] Batchelor S.N., Carr D., Coleman C.E., Fairclough L., Jarvis A. (2003). The photofading mechanism of commercial reactive dyes on cotton. Dye. Pigment..

[20] Kan C., Cheung H., Chan Q. (2016). A study of plasma-induced ozone treatment on the colour fading of dyed cotton. J. Clean. Prod..

[21] Anand S.C., Horrocks A.R., Anand S.C. (2016). Technical Fabric Structures—2. Knitted Fabrics. Handbook of Technical Textiles.

[22] Cai Y., Cui X., Rodgers J., Thibodeaux D., Martin V., Watson M., Pang S.-S. (2013). A Comparative Study of the Effects of Cotton Fibre Length Parameters on Modeling Yarn Properties. Text. Res. J..

[23] Martin P.C., Eyring M.B., Germer T.A., Zwinkels J.C., Tsai B.K. (2014). Microspectrophotometry. Experimental Methods in the Physical Sciences.

[24] Wiggins K., Davis E., Schennum C., Drummond P. (2008). An Investigation into Fibre Variation across Garments. Glob. Forensic Sci. Today.

